# Inflammatory Cells in Diffuse Large B Cell Lymphoma

**DOI:** 10.3390/jcm9082418

**Published:** 2020-07-28

**Authors:** Roberto Tamma, Girolamo Ranieri, Giuseppe Ingravallo, Tiziana Annese, Angela Oranger, Francesco Gaudio, Pellegrino Musto, Giorgina Specchia, Domenico Ribatti

**Affiliations:** 1 Department of Basic Medical Sciences, Neurosciences, and Sensory Organs, University of Bari Medical School, 70124 Bari, Italy; tiziana.annese@uniba.it; 2 IRCCS Istituto Tumori ’Giovanni Paolo II’, 70124 Bari, Italy; giroran@tiscalinet.it; 3 Pathology Section, Department of Emergency and Transplantation, University of Bari Medical School, 70124 Bari, Italy; giuseppe.ingravallo@uniba.it; 4 Section of Human Anatomy and Histology, Department of Emergency and Organ Transplantation, University of Bari, 70124 Bari, Italy; angelaoranger@yahoo.it; 5 Hematology Section, Department of Emergency and Transplantation, University of Bari Medical School, 70124 Bari, Italy; francesco.gaudio@uniba.it (F.G.); pellegrino.musto@uniba.it (P.M.); giorgina.specchia@uniba.it (G.S.)

**Keywords:** DLBCL, tumor microenvironment, tumor cells, T cells, neutrophils, NK cells, dendritic cells, macrophages

## Abstract

Diffuse large B cell lymphoma (DLBCL), known as the most common non-Hodgkin lymphoma (NHL) subtype, is characterized by high clinical and biological heterogeneity. The tumor microenvironment (TME), in which the tumor cells reside, is crucial in the regulation of tumor initiation, progression, and metastasis, but it also has profound effects on therapeutic efficacy. The role of immune cells during DLBCL development is complex and involves reciprocal interactions between tumor cells, adaptive and innate immune cells, their soluble mediators and structural components present in the tumor microenvironment. Different immune cells are recruited into the tumor microenvironment and exert distinct effects on tumor progression and therapeutic outcomes. In this review, we focused on the role of macrophages, Neutrophils, T cells, natural killer cells and dendritic cells in the DLBCL microenvironment and their implication as target for DLBCL treatment. These new therapies, carried out by the induction of adaptive immunity through vaccination or passive of immunologic effectors delivery, enhance the ability of the immune system to react against the tumor antigens inducing the destruction of tumor cells.

## 1. Introduction

### 1.1. Diffuse Large B Cell Lymphoma

Diffuse large B cell lymphoma (DLBCL) a neoplasm of large B-cells arranged in a diffuse pattern, is the most common form of non-Hodgkin’s lymphoma (NHL), accounting for about 49% of B cell cancers worldwide [1]. The median age of prevalence of DLBCL is the seventh decade, although it has been observed also in young adults and rarely in children with a mild male predominance [2]. In DLBCL affected patients a fast growing tumor mass develops in one or more lymph nodes and/or in extranodal sites. In relation to the extranodal sites, there are no limit on the organs in which the tumor could develop, although the gastrointestinal tract constitutes the more frequent primary tumor site [3].

The complex DLBCL classification has improved over time because the tumor includes heterogenic variants in relation to morphology, phenotype, genetic anomalies, prognosis and clinical characteristics (Table 1) [4]. About 50 years ago, the lymphomas were classified on the basis of morphological findings. Many aspects about the DLBCL were unknown so this cancer was called by various names. In 1969, the Rappaport classification system allowed to recognize DLBCL as diffuse histiocytic lymphoma [5]. As a consequence of the deepening of the immunological aspects related to the lymphomas, the development of new monoclonal antibodies and the implementation of molecular genetics are allowed to improve the acknowledgement of lymphomas, including DLBCL [6,7]. The high clinical and biological DLBCL heterogeneity is due to the concept that most of these lymphomas arise from germinal center B-cells at different stages of differentiation, in which recurrent genetic alterations contribute to the molecular pathogenesis of the disease [8].

### 1.2. Tumor Microenvironment Immune Cells

**Figure 1:**
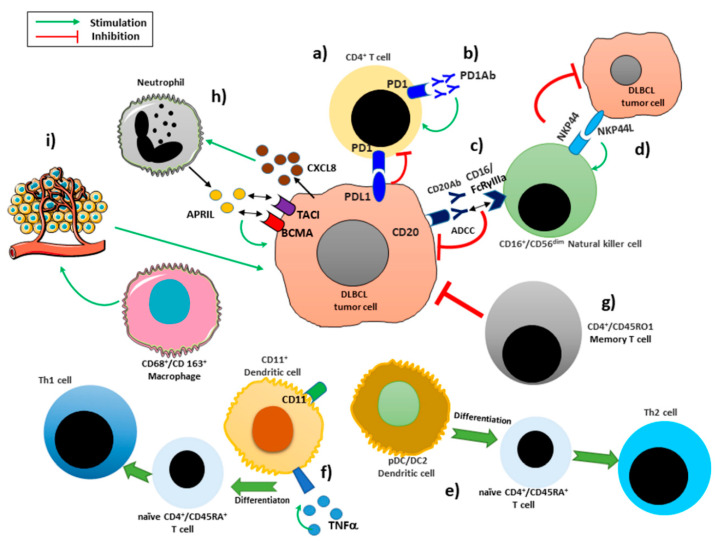
Some interactions involving the immune infiltrating cells in DLBCL microenvironment. The activation of PD-1 by its ligand PD-L1 induces the block of cell-cycle progression in CD4+ T cells (**a**). Antibodies blocking the interaction PD-1/PD-L1 restores the T cell mediated antitumor immune response (**b**). NK cells recognize CD20-Ab-coated cells by the type IIIA Fc receptor (FcRγIIIa; CD16a) and trigger NK cell-mediated ADCC, resulting in rapid NK-cell activation and degranulation (**c**). The activation of NKp44 improves the role of NK-cells against malignant cells (**d**). pDC/DC2 dendritic cells stimulate antigen naïve CD4+CD45RA+ T cells to differentiate into Th2 (**e**). DC1s are stimulated with tumor necrosis factor α (TNFα) acquire the capacity to induce the differentiation of naïve CD4+CD45RA+ T-cell to Th1 cells (**f**). Memory T characterized by a CD4+/CD45RO1 phenotype decrease the tumor proliferation rate (**g**). Tumor cells are able to recruit neutrophils considered as the major source of APRIL, through the release of CXCL8. APRIL binds to BCMA and TACI stimulating B cell maturation and differentiation and survival (**h**). CD163+ macrophages enhance immunosuppression and angiogenesis in tumor progression (**i**).

Cancers develop in complex tissue environments in which the tumor cells are surrounded by various types of cells, extracellular components and a vascular network that constitute the tumor microenvironment (TME) (Figure 1). The TME is involved in the regulation of tumor initiation, progression, and metastasis, but it also has profound effects on therapeutic efficacy [9]. The inflammatory microenvironment is an essential component of tumor microenvironment. Tissue-resident lymphocytes constitutively reside in non-lymphoid tissues, and generally do not re-circulate through blood [10]. Infiltrating lymphocytes have moved from the blood into a tissue. Tumor-infiltrating lymphocytes can recognize and kill cancer cells. The features of tumor infiltrating immune cells are correlated with the development and progression of cancer [11]. In cancer therapy, tumor-infiltrating lymphocytes are removed from a patient’s tumor, grown in large numbers, and then given back to the patient to help the immune system kill the cancer cells. In the recent years, many studies have demonstrated that the inflammatory microenvironment, growth factors, activated stroma, and DNA-damage-promoting agents, potentiates and/or promotes neoplastic risk. The balance of cytokines in any given tumor is critical for regulating the type and extent of inflammatory infiltrate that forms [12,13,14]. The abnormal expression of chemokines/chemokine receptors in DLBCL cells at mRNA and protein levels suggests a functional role for these chemokines in the interaction between lymphoma cells and tumor microenvironment [15]. These chemotactic interactions not only influence the biological properties of DLBCL cells, but also cause the tumor cells to increase immunosuppression, that further enhance tumor growth. Therefore, abnormal secretion of chemokines/chemokine receptors, which is earlier than imaging examination, may become effective means for predicting or targeting DLBCL [15]. Gupta et al. [16] found that the JAK/STAT pathway is strongly activated in DLBCL patients and the cytokines involved in the activation, included interelukin-2, -6 and -10 (IL-2, IL-6 and IL-10), and epidermal growth factor (EGF). In particular IL-10–induced JAK2 and STAT3 signaling [17]. It has also been showed that IL-10/IL-10 receptor (IL-10R) is the major cytokine involved in the activation of JAK2 in DLBCL cells. Hashwah and collaborators in a recent work showed that the IL-6 signaling pathway results activated in a subset of DLBCL patients especially of the ABC subtype with poor prognosis. Moreover, it seems that IL6 expression is correlated with the co-expression of active STAT3 and gp130 [18]. Cells resident in tumor inflammatory microenvironment, include macrophages, neutrophils, mast cells, myeloid derived suppressor cells, dendritic cells, natural killer cells, and T and B lymphocytes capable of producing an assorted array of cytokines, cytotoxic mediators, including reactive oxygen species, serine and cysteine proteases, matrix metalloproteinases (MMPs), membrane-perforating agents, and soluble mediators of cell killing, such as tumor necrosis factor alpha (TNF-α), interleukins and interferons (IFNs) [19,20]. A strong correlation between the activation of NF-κB or STAT3 has been found to operate by the infiltrating immune cells and the induction of pro-tumorigenic processes such as survival, proliferation, growth, angiogenesis, and invasion. On the other hand, the activation of NF-κB/STAT3 pathway stimulates the expression of immune/inflammatory cells attracting mediators, which sustain tumor-associated inflammation [21]. Understanding the tumor microenvironment allowed in the past decade the renewal of immunology and immunotherapy and the latter is now recognized as an important tool in the anti-tumor treatment. These new therapies carried out by induction of adaptive immunity through vaccination or passive of immunologic effectors delivery enhance the ability of the immune system to react against the tumor antigens inducing the destruction of tumor cells.

### 1.3. Epithelial Mesenchymal Transition (EMT) and Inflammatory Cells

Epithelial-mesenchymal transitions (EMTs), the acquisition of mesenchymal features from epithelial cells, are classified into three types: the first type occurs during embryonic development, the second type is associated with adult tissue regeneration, and the third type occurs in cancer progression. Approximately 90% of cancers exhibit some degree of EMT during their progression. After activation of EMT, tumor cells lose their epithelial features, including cell adhesion and polarity, reorganize their cytoskeleton, and acquire a mesenchymal morphology and the ability to migrate. Moreover, during EMT, a phenotypic switch has been observed with carcinomas that promotes the progression towards metastasis. Increasing literature data have emphasized that a link exists between cancer-associated EMT and chronic inflammation [22,23]. The link between EMT and immune recognition, and killing of cancer cells, is well-established and EMT contributes to immune escape of tumors. Recent reports have begun to investigate how the acquisition of mesenchymal features by carcinoma cells could also contribute to the development of an inflammatory and immunosuppressive tumor microenvironment in breast cancer [24,25,26,27,28] and metastatic non-small cell lung cancer [29].

## 2. Immune Infiltrating Cells

### 2.1. Macrophages

Tumor-associated macrophages (TAMs) derive from recruited monocytes and constitute a significant component of inflammatory infiltrates in neoplastic tissues. TAMs are CD68+ cells and have a dual role in neoplastic lesions. Macrophages possess remarkable plasticity and change their phenotype according to environmental stimuli. The M1 subset, which is involved in antitumor immunity and anti-angiogenesis and M2 CD163+ subset, have the opposing roles of enhancing immunosuppression and angiogenesis in tumor progression, and may be considered the two extremes of a large spectrum that can exert anti- and pro-tumoral activities [30,31]. It has been observed that macrophages are the major component in the microenvironment of DLBCL [32]. Studies on the gene expression profiles of DLBCL biopsy specimens have revealed the increased infiltration of macrophages into DLBCL stroma [33,34]. Patients with higher expression of CD68 in tumor microenvironment have a tendency to have poor treatment outcome of DLBCL [32]. In this context, an antibody against CD68 has been used as curative intent in a study involving DLBCL patients. The results of this trial did not show any significant correlation between the number of CD68+ cells and other clinical factors. Likewise, nor correlation was found between CD68+ cells in germinal center B-cell (GCB)/non-GCB immunophenotype or low/high Ki-67 percentage. Other data suggests the absence of significant correlation between the amount of CD68+ cells and progression-free survival or overall survival. These data have stated that the pro-tumorigenic effect of CD68+ macrophages has limited clinical relevance in DLBCL patients [35]. Although, CD68+ cell number seems don’t show any correlation with angiogenic response in both chemo-sensitive (GCB) and -resistant (ABC) DLBCL patients, it resulted increased in chemoresistant ones indicating an indirect role in stimulating angiogenesis [36,37]. While, the CD163/CD68 + cells ratio as predictive index for a poorer prognosis of DLBCL is still controversial, M2 macrophages seems to have an active role in tumor progression in DLBCL patient [38]. The increased CD163/CD68+ cells ratio and the content of CD163+ cells were linked to unfavorable prognosis [39]. Nam et al. evaluated the amount of M2 macrophages in R-CHOP (Rituximab, C: Cyclophosphamide, H: Doxorubicin Hydrochloride, O: Vincristine Sulfate, P: Prednisolone) treated DLBCL patients and they found that the higher number of CD163+ and CD163/CD68 + cells ratio was significantly associated with shorter overall survival. These data indicated that M2 could have a central role in the promotion of lymphoma function in DLBCL and in predicting poor clinical outcome [40]. The analysis of the tumor inflammatory microenvironment composition in DLBCL patients revealed the significant increased number of CD163+ cells in the ABC group of patients and a positive correlation between CD163+ cells and STAT3 expression in tumor cells [41]. The expression of STAT3 in tumor correlated with the increased angiogenesis in ABC group of patients [41].

### 2.2. Neutrophils

Studies concerning a large cohort of DLBCL patients concluded that patients with higher NLR (neutrophil to lymphocyte ratio) were more likely to have poorer prognosis than those with lower NLR [42]. Nowadays, NLR constitutes a prognostic value for patients with DLBCL [43]. Neutrophils are myeloid cells and account for approximately 50–70% of all white blood cells. Neutrophils represent the frontline defense against invading pathogens and the major component of the inflammatory process [44]. Tumor-infiltrating neutrophils TAN have been implicated in malignant development and progression, but mechanisms are still debated. TAN may acquire two different phenotypes: the N1 anti-tumorigenic phenotype or the N2 pro-tumorigenic phenotype and are classified, based on the state of their activation, cytokine expressed and effects on tumor growth [45]. The N1 phenotype is involved in cytotoxic activity against tumor cells and its immune profile is characterized by high levels of TNFα, CCL3, ICAM-1 and low levels of Arginase. The N2 neutrophils are characterized by upregulation of the chemokines CCL2, CCL3, CCL4, CCL8, CCL12, and CCL17, and CXCL1, CXCL2, IL-8/CXCL8 and CXCL16 and are involved in tumor growth, invasion, metastasis, angiogenesis and immunosuppression [46]. Specifically, N1 neutrophils are able to recruit CD8+T cells trough the production of a series of chemokines and cytokines including CCL3, CXCL9, CXCL10, IL-12, TNFα, granulocyte macrophage-colony stimulating factor (GM-CSF), and vascular endothelial growth factor (VEGF) [47]. It has been found that exists a crosstalk between CD4+, T helper 17 cells (Th17) and neutrophils mediated by the liberation of factors that include IL-17, CXCL8, TNFα, IFNγ and GM-CSF by Th17 and of CCL2, CCL20 by neutrophils [47,48,49]. Interestingly, a protein belonging to the TNF superfamily named A Proliferation-Inducing TNF Ligand (APRIL) co-stimulates B-cell activation and when overexpressed in mice induces B-cell neoplasia [50,51]. APRIL up-regulation has been observed in 46% of DLBCL patients where neutrophil it is revealed to be the main source of APRIL in tumor microenvironment. APRIL binds and accumulate by proteoglycans and its accumulation is correlated to the aggressiveness of lymphoma [52]. APRIL binds to BCMA (B cell maturation antigen) and TACI (transmembrane activator and CAML-interactor). B-cell maturation antigen (BCMA), in turn activates B-cell activating factor (BAFF) triggering to an intracellular signaling cascade JNK and NFkB mediated. The result is B cell maturation and differentiation into plasma cells [53]. The expression of BCMA has been expressed in B cell lymphoma highlighting its targeting in the potential use for the treatment of DLBCL patients [54,55,56]. Manfroi in a recent work demonstrated that in a significant fraction of DLBCL patients, tumor cells are able to recruit APRIL producing neutrophils through the release of CXCL8 in both GC and non-GC DLBCL subtypes that in turn induce DNA methylation and acetylation, crucial in DLBCL progression [57]. Moreover, Nie and coworkers suggest that also tumor-NETs (Neutrophil extracellular traps) is a useful prognostic biomarker in DLBCL. NETs formation implies the activation of Src, p38 and ERK signaling. NETs itself directly upregulates the Toll-like receptor 9 (TLR9) pathways in DLBCL and then NF-κB, STAT3 and p38 pathways promoting tumor progression. They also showed that disruption of NETs, blocking CXCL8-CXCR2 axis or inhibiting TLR9 could retard tumor progression in preclinical models [58].

### 2.3. Dendritic Cells

Dendritic cells (DCs) belong to antigen-presenting cells and their role is crucial in naïve T cells priming. The human circulating DCs population include two subsets that develop independently from a common precursor cell [59]. The first one is named as mDC/DC1 and includes CD11+ cells. When DC1s are stimulated with tumor TNF-α acquire the capacity to induce the differentiation of naïve CD4+CD45RA+ T-cell to Th1 cells. The second population, the pDC/DC2 has CD11c−/CD123 bright immunophenotype. The first one is named as mDC/DC1 and includes CD11+ cells. When DC1s are stimulated with TNF-α acquire the capacity to induce the differentiation of naïve CD4+CD45RA+ T-cell to Th1 cells. The second population, the pDC/DC2 has CD11c−/CD123 bright immunophenotype. These cells stimulate antigen naïve CD4+CD45RA+ T cells to differentiate into Th2 cells [60,61]. The presence of CD11+ DCs and granzyme B+ T cells into the tumors associated with denser S100 + cells and CD45RO+ T cells around the tumor edge correlated with a favorable prognosis [62]. A recent study investigated the role of CD11c positive DCs in DLBCL, indicating DCs and T-regulatory cells as mediators of anti-tumor factor production [63]. DCs are efficient antigen-presenting cells eliciting T-cell–mediated tumor destruction [64]. When pulsed with tumor-derived antigens or transduced with tumor antigen-encoding viruses or nucleic acids and then administered as a cellular vaccine, DCs promote protective and even therapeutic antitumor immunity in murine tumor models, providing a convincing basis for the clinical use of DCs in active vaccination strategies against human cancer [65]. Tumor-specific clonal immunoglobulin expressed by B-cell lymphomas have been used to pulse DCs in order to create and administrate a vaccine in patients with follicular B-cell lymphoma. All patients developed measurable antitumor cellular immune responses with cases of complete tumor regression or partial tumor regression [66]. In a pilot study in indolent B-NHL patients vaccination with autologous DCs, loaded with apoptotic and necrotic autologous tumor cells, has been used, which induced an increased natural killer (NK) cell activation parallel to a decrease in T-reg and induction of T- and B-cell antitumor responses [67]. The administration of antigenic or pro-inflammatory signals to improve DC engulfing, cross-presentation, and maturation, may increase the efficacy of DC-based vaccines [68]. Moreover, DCs transduced with RNA derived from lymphoma cell lines stimulate T-cell responses against HL-associated tumor antigens [69]. This latter technique is considered very interesting considered the minimal sample size required for the amplification of total tumor RNA.

### 2.4. T Lymphocytes

The adaptive immune cells influence the behavior of human tumors modulating tumor growth and invasion, and may constitute an important prognostic tool [70]. Evaluation of CD8+ cytotoxic T cells and CD45RO+ memory T cells in specific tumor regions could provide a useful information for the prediction of tumor recurrence and survival [71]. The shift to the T helper 2 (Th2) and T regulatory (Treg) immunosuppressive phenotypes correlates with the switch of cancer to an invasive form and confers the acquisition of immune response evasion properties [72]. T lymphocytes are component of DLBCL microenvironment. It is thought that their presence did not constitute only a residual element from the normal lymph node structure [73]. One of the first paper about the relation between T cell infiltration (TIL) and DLBCL claimed that in large B-cell lymphoma, a low percentage of Leu-2+ TILs correlated with a reduction in relapse free survival [74]. Other studies investigated the critical role exerted by T-lymphocytes in containing the malignant clone and in immunosurveillance and hypothesized that the tumor infiltrating CD4+ T cell may be even more important than CD8+ cells in determining patient outcome [75]. Memory T cells are involved in the downregulation of tumor proliferation rate [76], and DLBCL patients with less than 20% of infiltrating CD4+ cells have an inferior failure-free survival and overall survival [73]. Moreover, it was also shown that these cells are memory T cells characterized by a CD4+/CD45RO1 phenotype, and further studies showed that an increased number of activated memory CD4+ T cells infiltrating areas of B-cell lymphoma correlates with a lower proliferative rate of cells [73]. CD4^+^ follicular T-cells are partially defined by the high expression of Programmed cell death 1 (PD-1) and comprise both follicular helper (Tfh) T-cells and repressive (Tfr) T-cells [77]. PD-1, is an immune-inhibitory receptor belonging to B7 receptor family that when activated by its ligand PD-L1 induces the block of cell-cycle progression in T cells, and the inhibition of cytokine production [78]. Although, the high presence of PD1^+^ TIL is correlated with unfavorable prognosis it has been reported that in DLBCL patients the higher PD-1 expression on tumor-infiltrating lymphocytes predicts a favorable overall survival [79]. Among the TIL, a hi PD-1 and FoxP3^+^ cell populations have been described in DLBCL microenvironment and the number of PD-1^hi^ and FoxP3^+^ cells, as well as total CD4^+^ T-cells are associated with improved clinical outcome [80]. Preventing the interaction PD-1/PD-L1 by the immune-targeting of tumor cells using humanized antibodies against PD-1 or PD-L1 could restore the anti-tumor activity of the T cells [81]. The objective response rates to this therapy in patients with relapsed/refractory DLBCL remain of modest entity (10–36%) [82,83,84], depending on the high clinical and biological heterogeneity nature of DLBCL, as demonstrated by gene expression profiling and large-scale genomic analyses [8,85]. It could be useful to deepen the characterization of TIL in order to better understand or discovery the biological markers useful to select the patients adapt to anti PD1/PDL1 treatment. PD-L1 gene alterations are associated with response to PD-1 blockade in DLBCL and PD-L1 alterations have been used to identify a unique biological subset of DLBCL in which an endogenous anti-lymphoma immune response has been activated, and is associated with responsiveness to PD-1 blockade therapy [83].

#### PD-1/PD-L1 Blockade Therapy

**Figure 2:**
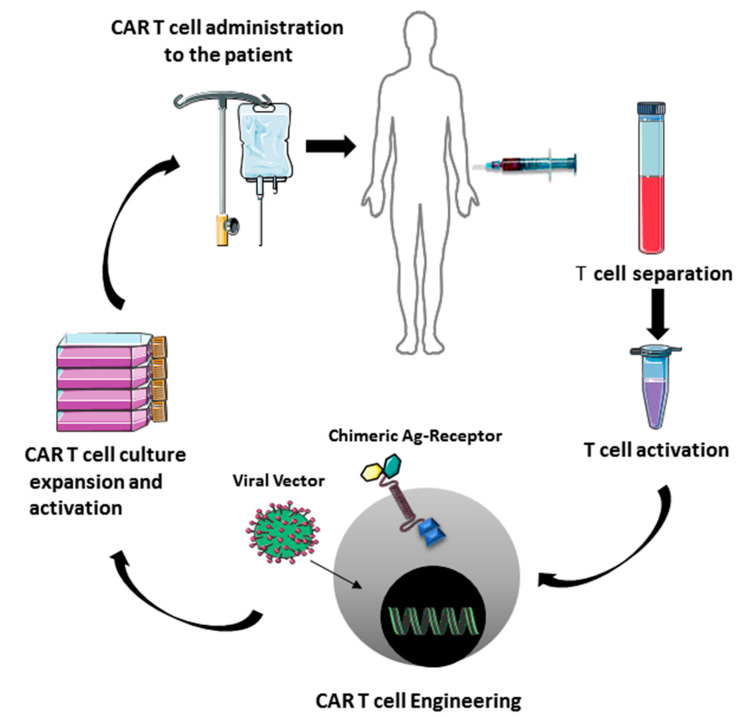
CAR-T cell immunotherapy process. T cells are separated and treated in order to obtain engineered T cells expressing CARs. The CAR-T cell are infused to the patient.

**Figure 3:**
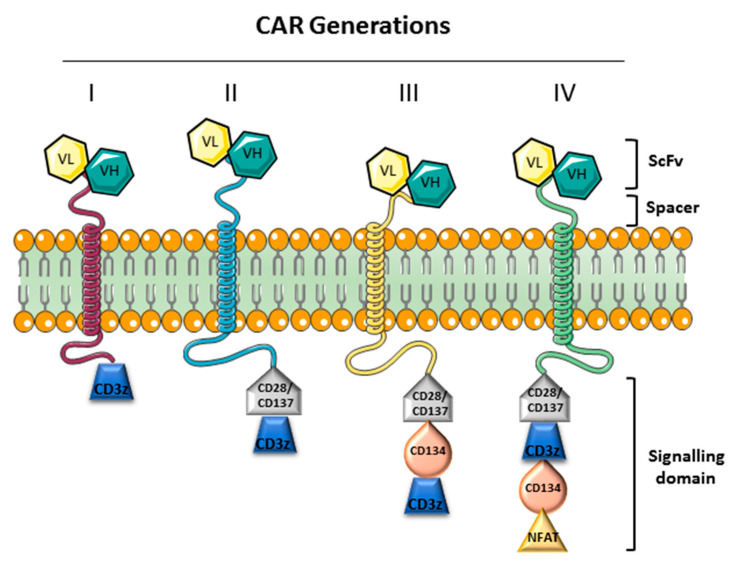
Schematic representation of CAR generation molecules.

Recently it was emerged that of PD-1/PD-L1 blockade therapy may have a beneficial influence on the efficacy of a recent emerging immunotherapy that utilize T lymphocytes [86,87]. This immunotherapy has been used in various diseases including hematological malignancies, solid tumors, autoimmune diseases, and allergic diseases such as asthma. The basis for this immunotherapy are the T cells that can be genetically manipulated in order to express a chimeric antigen receptor (CAR) other than their T cell receptor (TCR) (Figure 2). CAR induces the specific antigen targeting without the involvement of MHC system so bypassing the tumor cells immune evasion mechanism [88]. The CAR specificity has been obtained by the combination of B cell receptor derived and T cell receptor domains. CAR design has evolved over the years to enhance efficacy and safety in particular immunologic settings. It is derived by the fusion of three domains: The extracellular, the transmembrane and two intracellular domains, the costimulatory and the zeta chain domain. CAR extracellular domain does not comprise alpha and beta chains but is composed of single chain variable fragments (scFv), derived from heavy and light chain variable domains of the antibody. CAR must recruit endogenous downstream signaling molecules to transduce activating signal, but co-stimulation is provided in cis and in response to the same activating signal. Different generations of CARs can be distinguished (Figure 3).

The second-generation CAR-T cells have stimulating signaling domains (CD28, CD 137/4-1BB) responsible for T cell activation and expansion. Moreover, these domains stimulate the expansion of memory T cells and the survival of CAR-T cells [89]. The third generation of CARs the combination of multiple signaling domains (CD3z-CD28- CD134/OX40 or CD3z-CD28-CD137) enhanced the cytokine production and killing ability [90]. These third CARs generation have been used in the treating of lymphoma and colon cancer but the results of the few data available were comparable to the second generation [91,92]. The fourth generation of CARs are also named as TRUCKs (T cell redirected for universal cytokine-mediated killing) and have been obtained by the insertion of IL-12 to the construct of the second generation CARs. In this way, the activation of T cell is enhanced, as well as the recruitment the innate immunity, in order to eliminate antigen-negative cancer cells [93,94]. Increasingly, data on the therapeutic use of CARs against CD19 in hematological disease are emerging in recent years. CD19 is an integral membrane glycoprotein expressed by premature and mature B cells as well as on the majority of B cell malignancies. In this context in first Food and Drug Administration (FDA) and then European Medicines Agency (EMA), approved the use of Car T cells to overcome refractoriness and improve outcome when the conventional chemotherapy often fails in relapsed patients.

Axicabtagene ciloleucel (KTE-C19) is an immunotherapy treatment based on genetically modified autologous T cells in order to recognize the CD19 antigen used to treat adult patients with refractory/relapsed (r/r) DLBCL and primary mediastinal B cell lymphoma (PMBCL) after 2 or more systemic lines of therapy. Tisagenlecleucel has been approved. For pediatric and young adult patients affected by B cell acute lymphoblastic leukemia (ALL) and patients with r/r DLBCL tisagenlecleucel has been approved. Another CD19-directed CAR-T cell product still under investigation in TRANSCEND trial study (CTN02631044) is the lisocabtagene maraleucel JCAR017. This drug is composed of a well-defined ratio of CD4^+^ and CD8^+^ lymphocytes transduced with a lentiviral vector in order to express anti-CD19 scFv fused to CD137, the CD3-zeta, and a truncated form of the human epidermal growth factor receptor (EGFRt). EGFRt both facilitates the detection of the administered CARs and the promotion of their elimination through a cetuximab-induced ADCC response. The CD137 enhances both proliferation of T cells and antitumor activity [95,96].

Although, CAR-T cell therapy have shown encouraging results for patients who have no adequate treatment alternatives, it has also been associated with significant adverse effects, including tissue inflammation, neurotoxicity, hypoplasia in target cells, heart and pulmonary vasculature toxicity, cholangitis, injury to bile duct epithelial cells, and anaphylactic shock, which can be severe or lead to death [97,98,99]. Moreover, not all the treated patients respond to CAR-T cell therapy because of the loss of epitope or specific mutations in patients relapsing after CAR-T cells, but is working to reduce this problem [100,101].

### 2.5. Natural Killers Cells

Natural killer (NK) cells are a subclass of lymphocytes considered to be an important component of the immune system by controlling microbial infections and tumor progression [102]. NK are CD3^−^CD56^+^ cells able to recognize and kill malignant cells without previous sensitization, and include two major phenotypes on the basis of their level of CD56 expression: The mature cytotoxic CD16^+^CD56^dim^ (90% of circulating NK cells), and the less mature cytokine producing CD16^−^CD56^brigt^ cells, which reside predominantly in lymphoid tissue [103]. Although, they were discovered more than 40 years ago, due to their important role in acquired immune response, especially in the induction of memory for specific antigen for secondary immune response, NK cells have recently been attracting attention for their potential in immune-based therapies [104]. DLBCL patients showed lymphocytopenia involving the CD4^+^, CD8^+^ T, and NK cell subsets, but only NK cells number is correlated with induction treatment response and event free survival [105]. In many hematologic malignances, it has been observed that various mechanisms adopted by tumor cells escape from NK innate immune pressure, including the abnormal NK cytolytic functions [106]. In DLBCL the anti-CD20 monoclonal antibody rituximab constitute the keystone in the treatment of patients. Rituximab causes the elimination of CD20^+^ B cells by antibody-dependent cellular cytotoxicity (ADCC), complement-dependent cytotoxicity and direct induction of apoptosis [107]. In this context NK cells recognize CD20-Ab-coated target cells by the activating type IIIA Fc receptor (FcRγIIIa; CD16a) and trigger NK cell-mediated ADCC, resulting in rapid NK-cell activation and degranulation [108,109]. With the intent of NK cell activation as a strategy to improve the immunotherapy of DLBCL, authors studied NKp44, one of NCR receptors, which its activation improve its role against malignant cells [110]. The Increased expression of NKp44 was associated with lower values of LDH and earlier stages of DLBCL, hence, improvement of its function could constitute an approach of immunotherapy of DLBCL [110,111].

Although, research has mainly concentrated on the effect of PD-1 blockade on T cells, the recurrent deficits in major histocompatibility complex class I/II-associated antigen presentation in DLBCL cells suggested that the inhibition of PD-1 also involve additional mechanisms of action to that of cytotoxic T-cell-mediated killing in these lymphomas [112,113]. Moreover this defect enhance malignant B cells sensitivity to human CD3^−^CD56^+^ NK cells [114]. In this context, the therapeutic monoclonal antibodies in DLBCL enhance NK cytotoxicity against tumor cells [115]. Interestingly Vari et al described an unknown immune evasion mechanism in which is involved an alteration in the proportion of NK cells with a PD-1^hi^CD3^−^CD56^hi^CD16^-ve^ phenotype. Moreover, they hypothesized that the inhibition of NK cells occurs by PD-L1/PD-L2 expressing CD163^+^ monocyte/macrophages [116].

## 3. Concluding Remarks

Inflammation has been strongly correlated with cancer, implying a role for the inflammatory infiltrate to enhance the development of malignancies. Inflammatory cells establish a cross-talk with tumor cells, stromal cells and endothelial cells to create a complex microenvironment, essential for the survival and development of the malignancy. DLBCL is a disease characterized by a complex pathogenesis and behavior due to its clinical and biological heterogeneity but also to the TME composition and its interactions with neoplastic cells. In addition to current DLBCL classification criteria, and other prognostic markers, microenvironment evaluation constitutes a helpful instrument to better discriminate the groups of patients with worse prognosis and individuate new therapeutic approaches for the administration of personalized therapy. Although, the DLBCL biology knowledge has improved, the molecular mechanism, through the different elements of TME, regulates its aggressiveness has to be deepened and further studies with larger cohorts and longer follow-up have to be encouraged. Current frontline DLBCL therapy although fairly successful (70–80% remission rates with the standard R-CHOP chemotherapy regimen) is frequently followed by relapse (40% of cases within 2–3 years), with an often refractory DLBCL. Microenvironment-directed therapy represents important tools for the treatment of human lymphomas.

## Figures and Tables

**Table 1:** 2016 update of WHO classification of DLBCL: subtypes and related entities [4].

Diffuse large B-cell lymphoma, NOS	GCB versus ABC/non-GCB
	MYC and BCL2 double expressor
	CD5+
DLBCL subtypes	T-cell/histiocyte-rich large B-cell lymphoma
	Primary DLBCL of the central nervous system
	Primary cutaneous DLBCL, leg type
	EBV positive DLBCL, NOS
Other lymphomas of large B-cells	Primary mediastinal (thymic) large B-cell lymphoma
	Intravascular large B-cell lymphoma
	DLBCL associated with chronic inflammation
	Lymphomatoid granulomatosis
	ALK-positive DLBCL
	Plasmablastic lymphoma
	HHV8+ DLBCL, NOS
	Primary effusion lymphoma
Borderline cases	High-grade B-cell lymphoma, with MYC and BCL2 and/or BCL6 translocations
	High-grade B-cell lymphoma, NOS
	B-cell lymphoma, unclassifiable, with features intermediate between DLBCL and classical Hodgkin lymphoma

DLBCL: diffuse large B-cell lymphoma; ABC: activated B-cell like; GCB: germinal center B-cell like; HHV8: human herpesvirus 8; MYC: MYC proto-oncogene; NOS: not otherwise specified; EBV: Epstein-Barr Virus; ALK: Anaplastic lymphoma kinase; Bcl-2: B-cell lymphoma 2; Bcl-6: B-cell lymphoma 6; WHO: World Health Organization.

## References

[1] Campo E., Swerdlow S.H., Harris N.L., Pileri S., Stein H., Jaffe E.S. (2011). The 2008 WHO classification of lymphoid neoplasms and beyond: Evolving concepts and practical applications. Blood.

[2] Teras L.R., DeSantis C.E., Cerhan J.R., Morton L.M., Jemal A., Flowers C.R. (2016). 2016 US lymphoid malignancy statistics by World Health Organization subtypes. CA Cancer J. Clin..

[3] Ollila T.A., Olszewski A.J. (2018). Extranodal Diffuse Large B Cell Lymphoma: Molecular Features, Prognosis, and Risk of Central Nervous System Recurrence. Curr. Treat. Options Oncol..

[4] Swerdlow S.H., Campo E., Pileri S.A., Harris N.L., Stein H., Siebert R., Advani R., Ghielmini M., Salles G.A., Zelenetz A.D. (2016). The 2016 revision of the World Health Organization classification of lymphoid neoplasms. Blood.

[5] Swerdlow S.H. (2013). Lymphoma classification and the tools of our trade: An introduction to the 2012 USCAP Long Course. Mod. Pathol..

[6] Taylor C.R., Hartsock R.J. (2011). Classifications of lymphoma; reflections of time and technology. Virchows Arch..

[7] Pizzi M., Gazzola A., Mannu C., Pileri S.A., Sabattini E., Pileri S.A. (2014). The role of molecular biology in the diagnosis of lymphoid neoplasms. Front. Biosci..

[8] Schneider C., Pasqualucci L., Dalla-Favera R. (2011). Molecular pathogenesis of diffuse large B-cell lymphoma. Semin. Diagn. Pathol..

[9] Coussens L.M., Werb Z. (2002). Inflammation and cancer. Nature.

[10] Gasteiger G., Fan X., Dikiy S., Lee S.Y., Rudensky A.Y. (2015). Tissue residency of innate lymphoid cells in lymphoid and nonlymphoid organs. Science.

[11] Li Y.W., Qiu S.J., Fan J., Zhou J., Gao Q., Xiao Y.S., Xu Y.F. (2011). Intratumoral neutrophils: A poor prognostic factor for hepatocellular carcinoma following resection. J. Hepatol..

[12] Hanahan D., Coussens L.M. (2012). Accessories to the crime: Functions of cells recruited to the tumor microenvironment. Cancer Cell.

[13] Quail D.F., Joyce J.A. (2013). Microenvironmental regulation of tumor progression and metastasis. Nat. Med..

[14] Ranieri G., Patruno R., Lionetti A., Di Summa A., Mattioli E., Bufo P., Pellecchia A., Ribatti D., Zizzo N. (2005). Endothelial area and microvascular density in a canine non–Hodgkin’s lymphoma: An interspecies model of tumor angiogenesis. Leuk. Lymphoma.

[15] Shain K.H., Dalton W.S., Tao J. (2015). The tumor microenvironment shapes hallmarks of mature B–cell malignancies. Oncogene.

[16] Gupta M., Han J.J., Stenson M., Maurer M., Wellik L., Hu G., Ziesmer S., Dogan A., Witzig T.E. (2012). Elevated serum IL-10 levels in diffuse large B–cell lymphoma: A mechanism of aberrant JAK2 activation. Blood.

[17] Alas S., Bonavida B. (2003). Inhibition of constitutive STAT3 activity sensitizes resistant non–Hodgkin’s lymphoma and multiple myeloma to chemotherapeutic drug–mediated apoptosis. Clin. Cancer Res..

[18] Hashwah H., Bertram K., Stirm K., Stelling A., Wu C.T., Kasser S., Manz M.G., Theocharides A.P., Tzankov A., Muller A. (2019). The IL–6 signaling complex is a critical driver, negative prognostic factor, and therapeutic target in diffuse large B–cell lymphoma. EMBO Mol. Med..

[19] Kuper H., Adami H.O., Trichopoulos D. (2000). Infections as a major preventable cause of human cancer. J. Intern. Med..

[20] Wahl L.M., Kleinman H.K. (1998). Tumor-associated macrophages as targets for cancer therapy. J. Natl. Cancer Inst..

[21] Karin M. (2006). Nuclear factor–kappaB in cancer development and progression. Nature.

[22] Shinde A., Hardy S.D., Kim D., Akhand S.S., Jolly M.K., Wang W.H., Anderson J.C., Khodadadi R.B., Brown W.S., George J.T. (2019). Spleen Tyrosine Kinase-Mediated Autophagy Is Required for Epithelial-Mesenchymal Plasticity and Metastasis in Breast Cancer. Cancer Res..

[23] Hardy S.D., Shinde A., Wang W.H., Wendt M.K., Geahlen R.L. (2017). Regulation of epithelial–mesenchymal transition and metastasis by TGF-beta, P–bodies, and autophagy. Oncotarget.

[24] Libring S., Shinde A., Chanda M.K., Nuru M., George H., Saleh A.M., Abdullah A., Kinzer-Ursem T.L., Calve S., Wendt M.K. (2020). The Dynamic Relationship of Breast Cancer Cells and Fibroblasts in Fibronectin Accumulation at Primary and Metastatic Tumor Sites. Cancers.

[25] Shinde A., Libring S., Alpsoy A., Abdullah A., Schaber J.A., Solorio L., Wendt M.K. (2018). Autocrine Fibronectin Inhibits Breast Cancer Metastasis. Mol. Cancer Res..

[26] Shinde A., Paez J.S., Libring S., Hopkins K., Solorio L., Wendt M.K. (2020). Transglutaminase-2 facilitates extracellular vesicle-mediated establishment of the metastatic niche. Oncogenesis.

[27] Shinde A., Wilmanski T., Chen H., Teegarden D., Wendt M.K. (2018). Pyruvate carboxylase supports the pulmonary tropism of metastatic breast cancer. Breast Cancer Res..

[28] Wilmanski T., Zhou X., Zheng W., Shinde A., Donkin S.S., Wendt M., Burgess J.R., Teegarden D. (2017). Inhibition of pyruvate carboxylase by 1alpha,25–dihydroxyvitamin D promotes oxidative stress in early breast cancer progression. Cancer Lett..

[29] Uzunalli G., Dieterly A.M., Kemet C.M., Weng H.Y., Soepriatna A.H., Goergen C.J., Shinde A.B., Wendt M.K., Lyle L.T. (2019). Dynamic transition of the blood–brain barrier in the development of non-small cell lung cancer brain metastases. Oncotarget.

[30] Schoppmann S.F., Birner P., Stockl J., Kalt R., Ullrich R., Caucig C., Kriehuber E., Nagy K., Alitalo K., Kerjaschki D. (2002). Tumor-associated macrophages express lymphatic endothelial growth factors and are related to peritumoral lymphangiogenesis. Am. J. Pathol..

[31] Tamma R., Ruggieri S., Annese T., Simone G., Mangia A., Rega S., Zito F.A., Nico B., Ribatti D. (2018). Bcl6/p53 expression, macrophages/mast cells infiltration and microvascular density in invasive breast carcinoma. Oncotarget.

[32] Cai Q.C., Liao H., Lin S.X., Xia Y., Wang X.X., Gao Y., Lin Z.X., Lu J.B., Huang H.Q. (2012). High expression of tumor–infiltrating macrophages correlates with poor prognosis in patients with diffuse large B–cell lymphoma. Med. Oncol..

[33] Liu C., Sun C., Huang H., Janda K., Edgington T. (2003). Overexpression of legumain in tumors is significant for invasion/metastasis and a candidate enzymatic target for prodrug therapy. Cancer Res..

[34] Lenz G., Wright G., Dave S.S., Xiao W., Powell J., Zhao H., Xu W., Tan B., Goldschmidt N., Iqbal J. (2008). Stromal gene signatures in large-B–cell lymphomas. N. Engl. J. Med..

[35] Hasselblom S., Hansson U., Sigurdardottir M., Nilsson-Ehle H., Ridell B., Andersson P.O. (2008). Expression of CD68+ tumor-associated macrophages in patients with diffuse large B–cell lymphoma and its relation to prognosis. Pathol. Int..

[36] Marinaccio C., Ingravallo G., Gaudio F., Perrone T., Ruggieri S., Opinto G., Nico B., Maiorano E., Specchia G., Ribatti D. (2016). T cells, mast cells and microvascular density in diffuse large B–cell lymphoma. Clin. Exp. Med..

[37] Zizzo N., Patruno R., Zito F.A., Di Summa A., Tinelli A., Troilo S., Misino A., Ruggieri E., Goffredo V., Gadaleta C.D. (2010). Vascular endothelial growth factor concentrations from platelets correlate with tumor angiogenesis and grading in a spontaneous canine non-Hodgkin lymphoma model. Leuk. Lymphoma.

[38] Shen L., Li H., Shi Y., Wang D., Gong J., Xun J., Zhou S., Xiang R., Tan X. (2016). M2 tumour–associated macrophages contribute to tumour progression via legumain remodelling the extracellular matrix in diffuse large B–cell lymphoma. Sci. Rep..

[39] Marchesi F., Cirillo M., Bianchi A., Gately M., Olimpieri O.M., Cerchiara E., Renzi D., Micera A., Balzamino B.O., Bonini S. (2015). High density of CD68+/CD163+ tumour-associated macrophages (M2–TAM) at diagnosis is significantly correlated to unfavorable prognostic factors and to poor clinical outcomes in patients with diffuse large B–cell lymphoma. Hematol. Oncol..

[40] Nam S.J., Go H., Paik J.H., Kim T.M., Heo D.S., Kim C.W., Jeon Y.K. (2014). An increase of M2 macrophages predicts poor prognosis in patients with diffuse large B–cell lymphoma treated with rituximab, cyclophosphamide, doxorubicin, vincristine and prednisone. Leuk. Lymphoma.

[41] Tamma R., Ingravallo G., Gaudio F., Annese T., Albano F., Ruggieri S., Dicataldo M., Maiorano E., Specchia G., Ribatti D. (2020). STAT3, tumor microenvironment, and microvessel density in diffuse large B–cell lymphomas. Leuk. Lymphoma.

[42] Mu S., Ai L., Fan F., Qin Y., Sun C., Hu Y. (2018). Prognostic role of neutrophil–to–lymphocyte ratio in diffuse large B cell lymphoma patients: An updated dose–response meta–analysis. Cancer Cell Int..

[43] Stefaniuk P., Szymczyk A., Podhorecka M. (2020). The Neutrophil to Lymphocyte and Lymphocyte to Monocyte Ratios as New Prognostic Factors in Hematological Malignancies—A Narrative Review. Cancer Manag. Res..

[44] Sionov R.V., Fridlender Z.G., Granot Z. (2015). The Multifaceted Roles Neutrophils Play in the Tumor Microenvironment. Cancer Microenviron..

[45] Fridlender Z.G., Sun J., Kim S., Kapoor V., Cheng G., Ling L., Worthen G.S., Albelda S.M. (2009). Polarization of tumor–associated neutrophil phenotype by TGF-beta: “N1” versus “N2” TAN. Cancer Cell.

[46] Fridlender Z.G., Albelda S.M. (2012). Tumor-associated neutrophils: Friend or foe?. Carcinogenesis.

[47] Scapini P., Lapinet-Vera J.A., Gasperini S., Calzetti F., Bazzoni F., Cassatella M.A. (2000). The neutrophil as a cellular source of chemokines. Immunol. Rev..

[48] Schrub J.C., Courtois H., Vuillermet P., Viardot N., Mezaize D. (1978). Weight loss of obese patients and fatigue. Study of muscular performance and weight changes. Sem. Hop..

[49] Liang S.C., Long A.J., Bennett F., Whitters M.J., Karim R., Collins M., Goldman S.J., Dunussi-Joannopoulos K., Williams C.M., Wright J.F. (2007). An IL–17F/A heterodimer protein is produced by mouse Th17 cells and induces airway neutrophil recruitment. J. Immunol..

[50] Tecchio C., Scapini P., Pizzolo G., Cassatella M.A. (2013). On the cytokines produced by human neutrophils in tumors. Semin. Cancer Biol..

[51] Kimberley F.C., Medema J.P., Hahne M. (2009). APRIL in B–cell malignancies and autoimmunity. Results Probl. Cell Differ..

[52] Schwaller J., Schneider P., Mhawech-Fauceglia P., McKee T., Myit S., Matthes T., Tschopp J., Donze O., Le Gal F.A., Huard B. (2007). Neutrophil–derived APRIL concentrated in tumor lesions by proteoglycans correlates with human B–cell lymphoma aggressiveness. Blood.

[53] Yu G., Boone T., Delaney J., Hawkins N., Kelley M., Ramakrishnan M., McCabe S., Qiu W.R., Kornuc M., Xia X.Z. (2000). APRIL and TALL–I and receptors BCMA and TACI: System for regulating humoral immunity. Nat. Immunol..

[54] Cho S.F., Anderson K.C., Tai Y.T. (2018). Targeting B–Cell Maturation Antigen (BCMA) in Multiple Myeloma: Potential Uses of BCMA–Based Immunotherapy. Front. Immunol..

[55] Hagner P.R., Waldman M., Gray F.D., Yura R., Hersey S., Chan H., Zhang M., Boss I., Gandhi A.K. (2019). Targeting B-Cell Maturation Antigen (BCMA) with CC-93269, a 2+1 T Cell Engager, Elicits Significant Apoptosis in Diffuse Large B–Cell Lymphoma Preclinical Models. Blood.

[56] Friedman K.M., Garrett T.E., Evans J.W., Horton H.M., Latimer H.J., Seidel S.L., Horvath C.J., Morgan R.A. (2018). Effective Targeting of Multiple B–Cell Maturation Antigen–Expressing Hematological Malignances by Anti–B–Cell Maturation Antigen Chimeric Antigen Receptor T Cells. Hum. Gene Ther..

[57] Manfroi B., McKee T., Mayol J.F., Tabruyn S., Moret S., Villiers C., Righini C., Dyer M., Callanan M., Schneider P. (2017). CXCL–8/IL8 Produced by Diffuse Large B–cell Lymphomas Recruits Neutrophils Expressing a Proliferation–Inducing Ligand APRIL. Cancer Res..

[58] Nie M., Yang L., Bi X., Wang Y., Sun P., Yang H., Liu P., Li Z., Xia Y., Jiang W. (2019). Neutrophil Extracellular Traps Induced by IL8 Promote Diffuse Large B–cell Lymphoma Progression via the TLR9 Signaling. Clin. Cancer Res..

[59] Robinson S.P., Patterson S., English N., Davies D., Knight S.C., Reid C.D. (1999). Human peripheral blood contains two distinct lineages of dendritic cells. Eur. J. Immunol..

[60] O’Neill D.W., Adams S., Bhardwaj N. (2004). Manipulating dendritic cell biology for the active immunotherapy of cancer. Blood.

[61] Rissoan M.C., Soumelis V., Kadowaki N., Grouard G., Briere F., de Waal Malefyt R., Liu Y.J. (1999). Reciprocal control of T helper cell and dendritic cell differentiation. Science.

[62] Chang K.C., Huang G.C., Jones D., Lin Y.H. (2007). Distribution patterns of dendritic cells and T cells in diffuse large B–cell lymphomas correlate with prognoses. Clin. Cancer Res..

[63] Lee S., Kim D.H., Oh S.Y., Kim S.Y., Koh M.S., Lee J.H., Lee S., Kim S.H., Kwak J.Y., Pak M.G. (2017). Clinicopathologic significance of tumor microenvironment CD11c, and FOXP3 expression in diffuse large B–cell lymphoma patients receiving rituximab, cyclophosphamide, anthracycline, vincristine, and prednisone (R–CHOP) combination chemotherapy. Korean J. Intern. Med..

[64] Fong L., Engleman E.G. (2000). Dendritic cells in cancer immunotherapy. Annu. Rev. Immunol..

[65] Timmerman J.M., Levy R. (1999). Dendritic cell vaccines for cancer immunotherapy. Annu. Rev. Med..

[66] Hsu F.J., Benike C., Fagnoni F., Liles T.M., Czerwinski D., Taidi B., Engleman E.G., Levy R. (1996). Vaccination of patients with B–cell lymphoma using autologous antigen–pulsed dendritic cells. Nat. Med..

[67] Di Nicola M., Zappasodi R., Carlo–Stella C., Mortarini R., Pupa S.M., Magni M., Devizzi L., Matteucci P., Baldassari P., Ravagnani F. (2009). Vaccination with autologous tumor–loaded dendritic cells induces clinical and immunologic responses in indolent B–cell lymphoma patients with relapsed and measurable disease: A pilot study. Blood.

[68] Zappasodi R., Pupa S.M., Ghedini G.C., Bongarzone I., Magni M., Cabras A.D., Colombo M.P., Carlo-Stella C., Gianni A.M., Di Nicola M. (2010). Improved clinical outcome in indolent B–cell lymphoma patients vaccinated with autologous tumor cells experiencing immunogenic death. Cancer Res..

[69] Winkler C., Steingrube D.S., Altermann W., Schlaf G., Max D., Kewitz S., Emmer A., Kornhuber M., Banning-Eichenseer U., Staege M.S. (2012). Hodgkin’s lymphoma RNA-transfected dendritic cells induce cancer/testis antigen–specific immune responses. Cancer Immunol. Immunother..

[70] Galon J., Costes A., Sanchez-Cabo F., Kirilovsky A., Mlecnik B., Lagorce-Pages C., Tosolini M., Camus M., Berger A., Wind P. (2006). Type, density, and location of immune cells within human colorectal tumors predict clinical outcome. Science.

[71] Pages F., Kirilovsky A., Mlecnik B., Asslaber M., Tosolini M., Bindea G., Lagorce C., Wind P., Marliot F., Bruneval P. (2009). In situ cytotoxic and memory T cells predict outcome in patients with early–stage colorectal cancer. J. Clin. Oncol..

[72] Fridman W.H., Remark R., Goc J., Giraldo N.A., Becht E., Hammond S.A., Damotte D., Dieu-Nosjean M.C., Sautes-Fridman C. (2014). The immune microenvironment: A major player in human cancers. Int. Arch. Allergy Immunol..

[73] Ansell S.M., Stenson M., Habermann T.M., Jelinek D.F., Witzig T.E. (2001). Cd4+ T–cell immune response to large B–cell non-Hodgkin’s lymphoma predicts patient outcome. J. Clin. Oncol..

[74] Lippman S.M., Spier C.M., Miller T.P., Slymen D.J., Rybski J.A., Grogan T.M. (1990). Tumor–infiltrating T–lymphocytes in B–cell diffuse large cell lymphoma related to disease course. Mod. Pathol..

[75] Lauritzsen G.F., Weiss S., Dembic Z., Bogen B. (1994). Naive idiotype–specific CD4+ T–cells and immunosurveillance of B–cell tumors. Proc. Natl. Acad. Sci. USA.

[76] Jacob M.C., Favre M., Lemarc’Hadour F., Sotto M.F., Bonnefoix T., Sotto J.J., Bensa J.C. (1992). CD45RA expression by CD4 T lymphocytes in tumors invaded by B–cell non–Hodgkin’s lymphoma (NHL) or Hodgkin’s disease (HD). Am. J. Hematol..

[77] Ramiscal R.R., Vinuesa C.G. (2013). T–cell subsets in the germinal center. Immunol. Rev..

[78] Freeman G.J., Long A.J., Iwai Y., Bourque K., Chernova T., Nishimura H., Fitz L.J., Malenkovich N., Okazaki T., Byrne M.C. (2000). Engagement of the PD–1 immunoinhibitory receptor by a novel B7 family member leads to negative regulation of lymphocyte activation. J. Exp. Med..

[79] Hartmann S., Hansmann M.L. (2014). Large B–cell lymphoma rich in PD–1+ T–cells: An overlooked subtype of diffuse large B–cell lymphoma?. Am. J. Clin. Pathol..

[80] Ahearne M.J., Bhuller K., Hew R., Ibrahim H., Naresh K., Wagner S.D. (2014). Expression of PD–1 (CD279) and FoxP3 in diffuse large B–cell lymphoma. Virchows. Arch..

[81] Kiyasu J., Miyoshi H., Hirata A., Arakawa F., Ichikawa A., Niino D., Sugita Y., Yufu Y., Choi I., Abe Y. (2015). Expression of programmed cell death ligand 1 is associated with poor overall survival in patients with diffuse large B–cell lymphoma. Blood.

[82] Armand P., Engert A., Younes A., Fanale M., Santoro A., Zinzani P.L., Timmerman J.M., Collins G.P., Ramchandren R., Cohen J.B. (2018). Nivolumab for Relapsed/Refractory Classic Hodgkin Lymphoma After Failure of Autologous Hematopoietic Cell Transplantation: Extended Follow–Up of the Multicohort Single–Arm Phase II CheckMate 205 Trial. J. Clin. Oncol..

[83] Godfrey J., Tumuluru S., Bao R., Leukam M., Venkataraman G., Phillip J., Fitzpatrick C., McElherne J., MacNabb B.W., Orlowski R. (2019). PD–L1 gene alterations identify a subset of diffuse large B–cell lymphoma harboring a T–cell-inflamed phenotype. Blood.

[84] Chen R., Zinzani P.L., Fanale M.A., Armand P., Johnson N.A., Brice P., Radford J., Ribrag V., Molin D., Vassilakopoulos T.P. (2017). Phase II Study of the Efficacy and Safety of Pembrolizumab for Relapsed/Refractory Classic Hodgkin Lymphoma. J. Clin. Oncol..

[85] Pericart S., Tosolini M., Gravelle P., Rossi C., Traverse-Glehen A., Amara N., Franchet C., Martin E., Bezombes C., Laurent G. (2018). Profiling Immune Escape in Hodgkin’s and Diffuse large B–Cell Lymphomas Using the Transcriptome and Immunostaining. Cancers.

[86] Cao Y., Lu W., Sun R., Jin X., Cheng L., He X., Wang L., Yuan T., Lyu C., Zhao M. (2019). Anti-CD19 Chimeric Antigen Receptor T Cells in Combination With Nivolumab Are Safe and Effective Against Relapsed/Refractory B–Cell Non–hodgkin Lymphoma. Front. Oncol..

[87] Liu X., Ranganathan R., Jiang S., Fang C., Sun J., Kim S., Newick K., Lo A., June C.H., Zhao Y. (2016). A Chimeric Switch-Receptor Targeting PD1 Augments the Efficacy of Second–Generation CAR T Cells in Advanced Solid Tumors. Cancer Res..

[88] Fesnak A.D., June C.H., Levine B.L. (2016). Engineered T–cells: The promise and challenges of cancer immunotherapy. Nat. Rev. Cancer.

[89] Brudno J.N., Kochenderfer J.N. (2018). Chimeric antigen receptor T–cell therapies for lymphoma. Nat. Rev. Clin. Oncol..

[90] Marin V., Pizzitola I., Agostoni V., Attianese G.M., Finney H., Lawson A., Pule M., Rousseau R., Biondi A., Biagi E. (2010). Cytokine–induced killer cells for cell therapy of acute myeloid leukemia: Improvement of their immune activity by expression of CD33–specific chimeric receptors. Haematologica.

[91] Till B.G., Jensen M.C., Wang J., Qian X., Gopal A.K., Maloney D.G., Lindgren C.G., Lin Y., Pagel J.M., Budde L.E. (2012). CD20–specific adoptive immunotherapy for lymphoma using a chimeric antigen receptor with both CD28 and 4–1BB domains: Pilot clinical trial results. Blood.

[92] Morgan R.A., Yang J.C., Kitano M., Dudley M.E., Laurencot C.M., Rosenberg S.A. (2010). Case report of a serious adverse event following the administration of T cells transduced with a chimeric antigen receptor recognizing ERBB2. Mol. Ther..

[93] Chmielewski M., Abken H. (2015). TRUCKs: The fourth generation of CARs. Expert Opin. Biol. Ther..

[94] Sha H.H., Wang D.D., Yan D.L., Hu Y., Yang S.J., Liu S.W., Feng J.F. (2017). Chimaeric antigen receptor T–cell therapy for tumour immunotherapy. Biosci. Rep..

[95] Sommermeyer D., Hudecek M., Kosasih P.L., Gogishvili T., Maloney D.G., Turtle C.J., Riddell S.R. (2016). Chimeric antigen receptor-modified T cells derived from defined CD8+ and CD4+ subsets confer superior antitumor reactivity in vivo. Leukemia.

[96] Milone M.C., Fish J.D., Carpenito C., Carroll R.G., Binder G.K., Teachey D., Samanta M., Lakhal M., Gloss B., Danet-Desnoyers G. (2009). Chimeric receptors containing CD137 signal transduction domains mediate enhanced survival of T cells and increased antileukemic efficacy in vivo. Mol. Ther..

[97] Neelapu S.S., Tummala S., Kebriaei P., Wierda W., Gutierrez C., Locke F.L., Komanduri K.V., Lin Y., Jain N., Daver N. (2018). Chimeric antigen receptor T–cell therapy—assessment and management of toxicities. Nat. Rev. Clin. Oncol..

[98] Morgan R.A., Chinnasamy N., Abate-Daga D., Gros A., Robbins P.F., Zheng Z., Dudley M.E., Feldman S.A., Yang J.C., Sherry R.M. (2013). Cancer regression and neurological toxicity following anti–MAGE–A3 TCR gene therapy. J. Immunother..

[99] Maus M.V., Haas A.R., Beatty G.L., Albelda S.M., Levine B.L., Liu X., Zhao Y., Kalos M., June C.H. (2013). T cells expressing chimeric antigen receptors can cause anaphylaxis in humans. Cancer Immunol. Res..

[100] Gardner R., Wu D., Cherian S., Fang M., Hanafi L.A., Finney O., Smithers H., Jensen M.C., Riddell S.R., Maloney D.G. (2016). Acquisition of a CD19–negative myeloid phenotype allows immune escape of MLL–rearranged B–ALL from CD19 CAR–T–cell therapy. Blood.

[101] Orlando E.J., Han X., Tribouley C., Wood P.A., Leary R.J., Riester M., Levine J.E., Qayed M., Grupp S.A., Boyer M. (2018). Genetic mechanisms of target antigen loss in CAR19 therapy of acute lymphoblastic leukemia. Nat. Med..

[102] Artis D., Spits H. (2015). The biology of innate lymphoid cells. Nature.

[103] Morvan M.G., Lanier L.L. (2016). NK cells and cancer: You can teach innate cells new tricks. Nat. Rev. Cancer.

[104] Cheng M., Chen Y., Xiao W., Sun R., Tian Z. (2013). NK cell–based immunotherapy for malignant diseases. Cell Mol. Immunol..

[105] Plonquet A., Haioun C., Jais J.P., Debard A.L., Salles G., Bene M.C., Feugier P., Rabian C., Casasnovas O., Labalette M. (2007). Peripheral blood natural killer cell count is associated with clinical outcome in patients with aaIPI 2–3 diffuse large B–cell lymphoma. Ann. Oncol..

[106] Baier C., Fino A., Sanchez C., Farnault L., Rihet P., Kahn-Perles B., Costello R.T. (2013). Natural killer cells modulation in hematological malignancies. Front. Immunol..

[107] Browning J.L. (2006). B cells move to centre stage: Novel opportunities for autoimmune disease treatment. Nat. Rev. Drug Discov..

[108] Borghaei H., Smith M.R., Campbell K.S. (2009). Immunotherapy of cancer. Eur. J. Pharmacol..

[109] Sconocchia G., Titus J.A., Segal D.M. (1997). Signaling pathways regulating CD44–dependent cytolysis in natural killer cells. Blood.

[110] Essa E.S., Tawfeek G.A., El Hassanin S.A., Emara K.G.M. (2018). Modulation the expression of natural killer cell activating receptor (NKp44) in the peripheral blood of diffuse large B–cell lymphoma patients and the correlation with clinic pathological features. Clin. Immunol..

[111] Cheng M., Ma J., Chen Y., Zhang J., Zhao W., Zhang J., Wei H., Ling B., Sun R., Tian Z. (2011). Establishment, characterization, and successful adaptive therapy against human tumors of NKG cell, a new human NK cell line. Cell Transpl..

[112] Jiang Y., Li Y., Zhu B. (2015). T–cell exhaustion in the tumor microenvironment. Cell Death Dis..

[113] Challa-Malladi M., Lieu Y.K., Califano O., Holmes A.B., Bhagat G., Murty V.V., Dominguez-Sola D., Pasqualucci L., Dalla-Favera R. (2011). Combined genetic inactivation of beta2–Microglobulin and CD58 reveals frequent escape from immune recognition in diffuse large B–cell lymphoma. Cancer Cell.

[114] Zaretsky J.M., Garcia-Diaz A., Shin D.S., Escuin-Ordinas H., Hugo W., Hu-Lieskovan S., Torrejon D.Y., Abril-Rodriguez G., Sandoval S., Barthly L. (2016). Mutations Associated with Acquired Resistance to PD–1 Blockade in Melanoma. N. Engl. J. Med..

[115] Arpon D.R., Gandhi M.K., Martin J.H. (2014). A new frontier in haematology—combining pharmacokinetic with pharmacodynamic factors to improve choice and dose of drug. Br. J. Clin. Pharmacol..

[116] Vari F., Arpon D., Keane C., Hertzberg M.S., Talaulikar D., Jain S., Cui Q., Han E., Tobin J., Bird R. (2018). Immune evasion via PD-1/PD-L1 on NK cells and monocyte/macrophages is more prominent in Hodgkin lymphoma than DLBCL. Blood.

